# Low-Dose Fluvastatin Prevents the Functional Alterations of Endothelium Induced by Short-Term Cholesterol Feeding in Rabbit Carotid Artery

**DOI:** 10.1100/2012/671728

**Published:** 2012-04-01

**Authors:** Gulnur Sevin, Yasemin Delen Akcay, Gonen Ozsarlak-Sozer, Mukadder Yasa

**Affiliations:** ^1^Department of Pharmacology, Faculty of Pharmacy, Ege University, Bornova, 35100 Izmir, Turkey; ^2^Department of Biochemistry, Faculty of Medicine, Ege University, 35100 Izmir, Turkey

## Abstract

3-Hydroxy-3-methylglutaryl coenzyme A (HMG-CoA) reductase inhibitors, commonly known as statins, are the medical treatment of choice for hypercholesterolemia. In addition to lowering serum-cholesterol levels, statins appear to promote pleiotropic effects that are independent of changes in serum cholesterol. In this study, we investigated the effects of low-dose fluvastatin on antioxidant enzyme activities (superoxide dismutase, SOD; catalase), total nitrite/nitrate levels, and vascular reactivity in 2% cholesterol-fed rabbits. This diet did not generate any fatty streak lesions on carotid artery wall. However, SOD activity significantly increased with cholesterol feeding whereas the catalase activities decreased. The levels of nitrite/nitrate, stable products of NO degradation, diminished. Moreover, dietary cholesterol reduced vascular responses to acetylcholine, but contractions to serotonin were augmented. Fluvastatin treatment abrogated the cholesterol-induced increase in SOD, increased the levels of nitric oxide metabolites in tissue, and restored both the impaired vascular responses to acetylcholine and the augmented contractile responses to serotonin without affecting plasma-cholesterol levels. Phenylephrine contractions and nitroglycerine vasodilatations did not change in all groups. This study indicated that fluvastatin treatment performed early enough to improve impaired vascular responses may delay cardiovascular complications associated with several cardiovascular diseases.

## 1. Introduction

Oxidative stress and endothelial dysfunction in the coronary and peripheral circulation have important prognostic implications for subsequent cardiovascular events [1]. Chronic elevations in serum cholesterol are often associated with an impaired endothelium-dependent vasodilation before the appearance of any ultrastructural change in the vessel wall of patients with risk for atherosclerosis [2]. This effect of hypercholesterolemia is due to the combination of increased oxidative stress leading to a reduced bioavailability of NO, impairment of the turnover rate of eNOS, and augmented levels of circulating asymmetric dimethyl arginine (ADMA, an endogenous inhibitor of eNOS) [3]. In order to protect tissues from the damaging effects of oxidative stress, the organism possesses enzymatic and nonenzymatic antioxidant systems [4]. It has been suggested that a chronic imbalance between formation of reactive oxygen species (ROS) and antioxidant capacity characterizes many disease states, such as atherosclerosis, carcinogenesis, and reperfusion injury [5, 6]. In opposition to endothelium-dependent relaxation responses, the vascular smooth muscle responsiveness is controversial in experimental hypercholesterolemia [7, 8].

HMG-CoA reductase inhibitors (statins) reduce cholesterol levels and prevent cardiovascular morbidity and mortality [9]. These drugs also exert benefits not related to the reduction of cholesterol levels; these are known as pleiotropic effects [10]. Several studies have demonstrated that independent of lipid lowering effects, fluvastatin has an antioxidant effect, decreases expression of adhesion molecules in monocytes and leukocyte-endothelium adherence responses, inhibits cholesterol esterification and accumulation in macrophages, along with effects on smooth muscle cell proliferation and migration [11]. Up to date there is no evidence that HMG-CoA reductase inhibitors exert protective effects on early-stage vascular functions with a high-cholesterol diet.

The present study aims to investigate the potential effects of fluvastatin, at a dose insufficient to cause the lipid-lowering effect, on antioxidant systems (superoxide dismutase: SOD, catalase) and nitrite/nitrate levels and the early-stage vascular functions in rabbits fed with 2% cholesterol-enriched diet for 4 weeks.

## 2. Materials and Methods

### 2.1. Materials

Cholesterol was purchased from Fluka (Buchs, Switzerland). Drugs for functional studies such as acetylcholine hydrochloride, phenylephrine hydrochloride, 5-hydroxytryptamine creatinine sulphate, N^*ω*^-nitro-L-arginine, and indomethacin were purchased from Sigma Chemical Co., (St. Louis, Mo, USA). Nitroglycerine solution was obtained from Merck, Sharp, and Dohme (Munich, Germany) and sodium pentobarbital from Psyphac (Brussels, Belgium).

### 2.2. Animals and Study Design

Animals were obtained from the Animal Breeding Facility of the Faculty of Pharmacy. The study had been approved by Animals Ethics Committee of the Faculty of Pharmacy of Ege University. White rabbits of either sex (*n* = 15) were divided into 3 groups. Control group was given a regular rabbit chow; cholesterol group was fed a regular rabbit chow plus 2% cholesterol; fluvastatin group received regular rabbit chow plus 2% cholesterol and fluvastatin (2 mg/kg/day, p.o.). Animals in all treatment groups consumed 100 g of chow daily. Each rabbit was kept in a separate cage and allowed access to tap water *ad libitum*. Each treatment was continued for 4 weeks. Weights were recorded every day. Blood samples were drawn from the central ear artery at the beginning and the end of the study to measure plasma cholesterol levels. Rabbits were sacrificed by an overdose of sodium pentobarbital (100 mg/kg) at the end of the feeding period. The carotid arteries were excised and dissected free of adhering adipose and connective tissue. Left carotid artery was cut into 3 mm rings for organ bath studies, and the right carotid artery was used for morphological and biochemical experiments. Tissue samples after being frozen under liquid nitrogen were stored at −80°C prior to analysis. On the day of analysis, the proximal carotid artery was cut into transverse sections and stained with ORO (Oil Red O) for morphometric analysis.

### 2.3. Biochemical Analyses

#### 2.3.1. Cholesterol Measurements

Plasma total cholesterol levels were determined with commercially available enzyme kits (Lieberman-Burchard, G-200 Cholesterol kit, Gökhan Lab. Tic. A.Ş. Izmir, Turkey). These levels were expressed as mmol/L.

#### 2.3.2. Measurements in Tissue Samples

The right carotid arteries were homogenized in phosphate buffer saline (pH = 7.4) in a ratio of 1/10 (w/v) and centrifuged at 600 g for 10 min; the supernatant was used for SOD, catalase assay, and measurement of nitrite/nitrate levels. SOD and catalase activities were determined as described previously [12]. Protein concentrations were measured using bovine-serum albumin as a standard by the method of Lowry et al. [13]. Nitrite levels were determined by a colorimetric method based on the Sozmen et al. [14]. Nitrate levels were measured by the enzymatic reduction of nitrate to nitrite by nitrate reductase from *Aspergillus* sp., in the presence of NADPH [15]. Oxidation of NADPH was recorded as the decrease in absorbance at 340 nm. Sodium nitrite and nitrate solutions were used for standard measurements. Tissue nitrate, nitrite, and total nitrite/nitrate levels were expressed as nmol/mg protein.

### 2.4. Organ Bath Experiments

Carotid artery rings were mounted on stainless steel wires connected to force transducers and placed in organ chambers for isometric tension recording and examined for vascular reactivity.

Studies were performed in organ chambers filled with 25 mL Kreb's physiological salt solution (PSS) with the following composition (mM): NaCl, 118.0; KCl, 4.7; CaCl_2_, 2.5; KH_2_PO_4_, 1.2; MgSO_4_, 1.2; NaHCO_3_, 25.0; glucose, 11.1 and equilibrated at 37°C with 95% O_2_-5% CO_2_ gas mixture. Tension was measured isometrically with a Grass FT 3 force transducer and recorded by IOSlab software package (IOS lab version 3.23 MS8, EMKA Technologies, Paris, France) using a 80486-based microcomputer (IBM PS/1, U.K.). Passive tension was gradually increased and adjusted to 7 g, which brings both control and atherosclerotic carotid artery to the optimal length for isometric contraction [16]. The segments were then allowed to equilibrate for 45 min prior to experimentation. During the equilibration period, the bath solution was changed every 15 min. At the end of this period tissues were contracted with 80 mM KCl to determine the contractility. Then, tissues were washed out three times and acetylcholine-induced endothelium-dependent, vasorelaxant responses resulting from the release of nitric oxide were tested. For this purpose, carotid artery rings were contracted with phenylephrine (10^−6 ^M), and during plateau contraction, acetylcholine (ACh) was added in a cumulative manner (10^−9 ^M–10^−4 ^M). Tissues which relaxed by more than 45% of the initial contraction were accepted as endothelium intact and washed out three times with PSS, recontracted with phenylephrine (10^−6 ^M), and then exposed to cumulative concentrations of nitroglycerine (NTG) (10^−9 ^M × 10^−5 ^M). Later, the tissues were washed out three times and treated with cumulative concentrations of phenylephrine (10^−9 ^M–10^−4 ^M) and serotonin (5-HT) (10^−9 ^M × 10^−5 ^M). Each agonist was washed out by changing the bath solution three times within 30 min before addition of the next agonist. Thereafter, the concentration-response curves of phenylephrine (10^−9 ^M–10^−4 ^M) and 5-HT (10^−9 ^M × 10^−5 ^M) were obtained in arterial rings following incubation of tissues with N^*ω*^-nitro-L-arginine (LNA; 10^−4 ^M, 30 min), a competitive inhibitor of constitutive and inducible nitric oxide synthase (NOS) isoforms, cyclooxygenase inhibitor indomethacin (INDO; 10^−5 ^M, 30 min) [17].

### 2.5. Statistical Analysis

All values are given as means ± S.E.M. The number of experiments refers to the number of animals. ACh- and NTG-induced relaxations were normalized to the phenylephrine precontractions and expressed as percentage. The negative logarithm of concentration (−log EC_50_ or pD_2_) that produced half of the maximal effect (*E*_max⁡_) of that agonist was calculated. Prism 3.02 (GraphPad Software, USA) was used for this purpose. All data were analysed by using the SPSS/PC^+^ package (SPSS, Chicago, Ill, USA) programme. The statistical comparisons between the groups were tested using one-way analysis of variance (ANOVA) followed by Bonferroni for multiple comparisons. The differences between tensions developed to N^*ω*^-nitro-L-arginine (LNA) and indomethacin (INDO) in the groups were assessed by Wilcoxon paired signed ranks test. Statistical significance was accepted at the 0.05 level of probability.

## 3. Results

### 3.1. Animals and Plasma-Cholesterol Levels

The body weights of animals were not different either from each other at the beginning and the end of the experimental protocol or between the groups (Table 1). Initial plasma cholesterol levels were not significantly different among the three groups. Cholesterol feeding increased plasma cholesterol levels at the end of 4 weeks and fluvastatin treatment (2 mg/kg/day) did not lower the plasma cholesterol levels (Table 1).

### 3.2. Morphological Alterations of Carotid Artery

**Figure 1:**
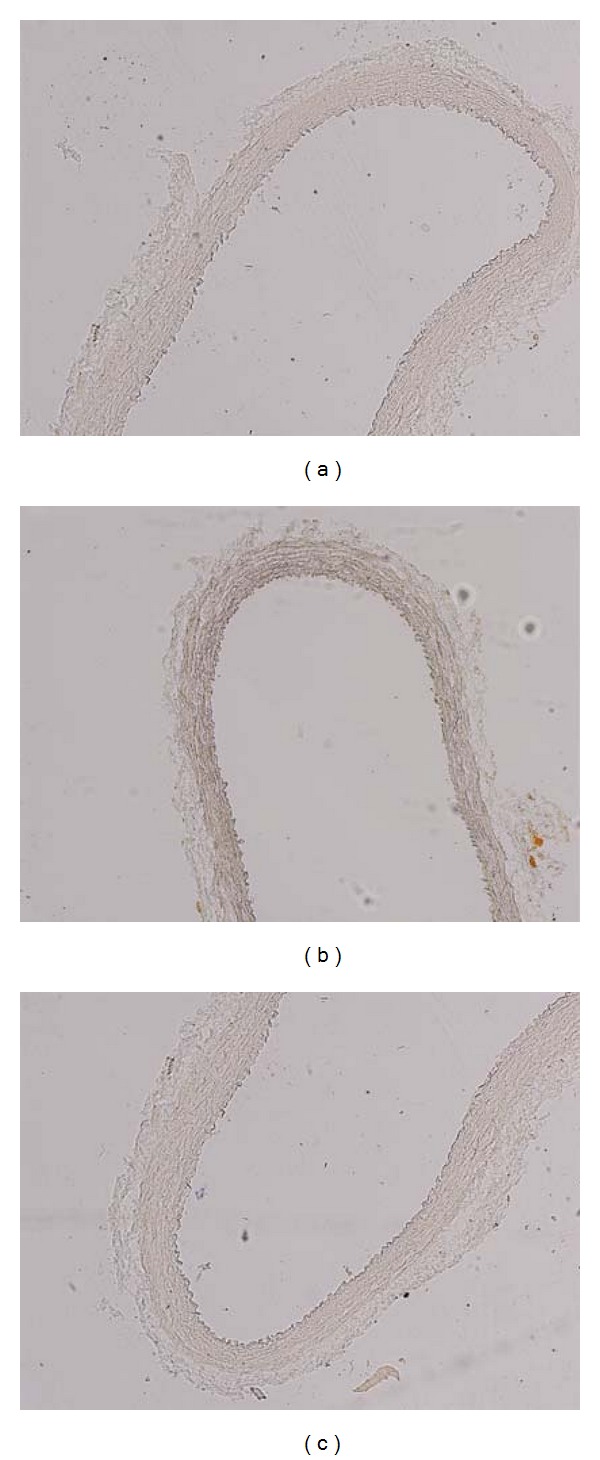
Photomicrographs of the proximal common carotid artery of the right side staining with Red-Oil. Original magnification ×100. Carotid artery from (a) control group; (b) cholesterol group; (c) cholesterol + fluvastatin group.

Lipid infiltration was not observed in carotid artery sections of cholesterol and cholesterol diet plus fluvastatin groups after staining with ORO (Oil Red O) (Figure 1).

### 3.3. Antioxidant Enzyme Activities in Carotid Tissues

While tissue catalase activity was decreased, the SOD activity was increased at the end of the 4 week cholesterol feeding period. However, treatment with fluvastatin did not affect the catalase activity but lowered the increased activity of SOD in cholesterol group (Table 2). Tissue NO metabolites (NO_2_^−^ and NO_3_^−^) were also determined in carotid artery tissues. Cholesterol feeding significantly decreased total nitrite/nitrate levels and fluvastatin treatment reversed the decrease in nitrite/nitrate levels (Table 2).

### 3.4. Vascular Reactivity

**Figure 2:**
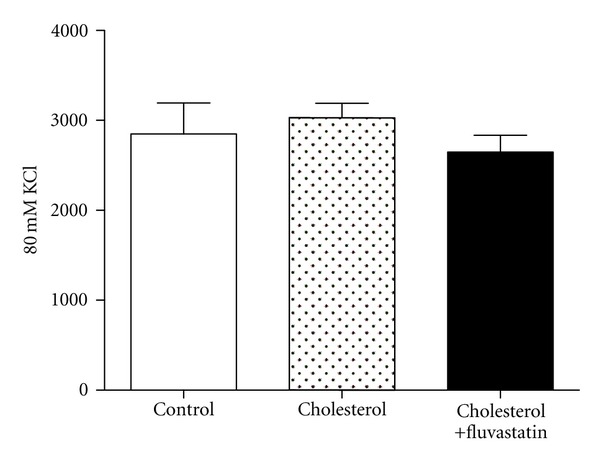
80 mM KCl-induced contractions in carotid arteries from all groups. Data shown in bar graphs are expressed as mean ± S.E.M.

**Figure 3:**
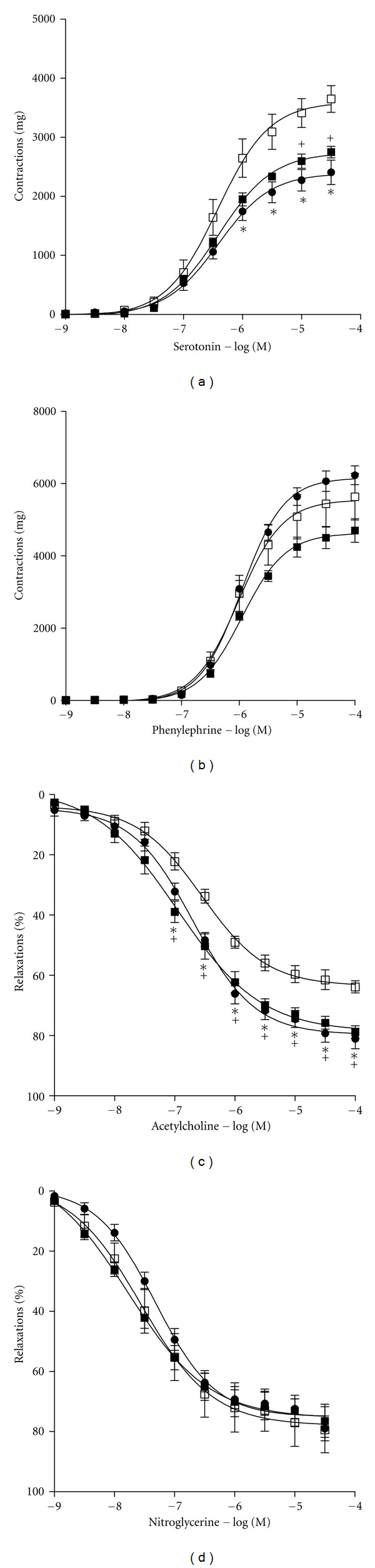
The effect of cholesterol feeding and fluvastatin treatment on vascular contractions to serotonin (a) and phenylephrine (b) and relaxations to acetylcholine (c) and nitroglycerine (d) in isolated rabbit carotid artery. Rings of carotid arteries from control (•) (*n* = 5), cholesterol (□) (*n* = 5), and cholesterol + fluvastatin (■) (*n* = 5) groups. Values are expressed as means ± S.E.M. Cumulative dose-response curves for ACh and NTG precontracted with 10^-6 ^M phenylephrine. *P* < 0.01 compared with control versus cholesterol (*) and cholesterol versus cholesterol + fluvastatin (^+^) groups, ANOVA followed by Bonferroni *post hoc* test.

**Figure 4:**
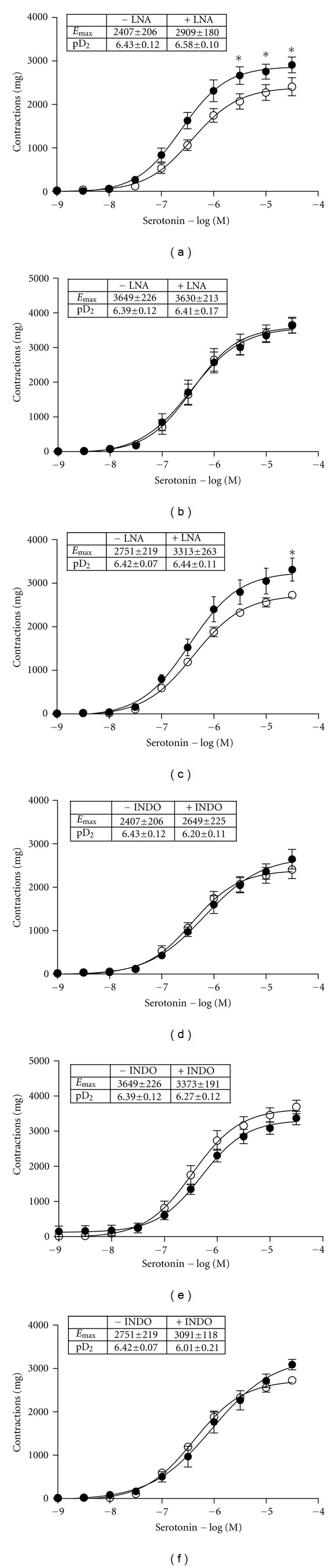
The effects of inhibition of nitric oxide synthesis by LNA and cyclooxygenase inhibition by INDO on the contractile responses to serotonin in isolated rabbit carotid artery. Figures from control group (a, d), cholesterol group (b, e), and cholesterol + fluvastatin group (c, f). Ring presentations of carotid arteries from −LNA or −INDO (◯) (*n* = 5) and +LNA or +INDO (•) (*n* = 5) in the groups. Values are expressed as means ± S.E.M. *P < 0.05 compared with −LNA and +LNA or −INDO and +INDO in the groups, Wilcoxon paired signed ranks test.

80 mM KCl-induced contractions were affected by neither cholesterol feeding nor fluvastatin treatment (Figure 2). 5-HT and phenylephrine induced concentration-dependent contractions in the carotid artery rings of all groups (Figure 3). Cholesterol feeding caused an increase of 5-HT contractions and fluvastatin treatment normalized the augmented vascular responses to this agonist. Besides, in rings from fluvastatin group, in the presence of LNA, 5-HT-induced contractions were significantly increased, indicating the contribution of endothelial nitric oxide to the effect of fluvastatin (Figures 4(a), 4(b), and 4(c)). However, the contractile responses of this agonist were not changed in the presence of indometacin (Figures 4(d), 4(e), and 4(f)). On the other hand, the *E*_max⁡_ and pD_2_ values of phenylephrine were not changed in all groups (Figure 3).

ACh and NTG induced concentration-dependent relaxations in carotid artery rings from all groups (Figure 3). In the cholesterol group, relaxations to ACh were significantly impaired as compared to control group and treatment with fluvastatin significantly restored these reduced relaxations. Cholesterol feeding and fluvastatin treatment did not affect the *E*_max⁡_ and pD_2_ values of nitroglycerine (Figure 3).

## 4. Discussion

It has been demonstrated that the preventive effect of statins on coronary events is not only attributed to cholesterol-lowering, but also to various effects on the vascular wall, which include improved endothelial function as well as antioxidant and anti-inflammatory activity [18–20]. In this study, pleiotropic effects of fluvastatin on alterations in vascular reactivity and antioxidant enzyme activities and total nitrite/nitrate levels were examined by assessing the responses of carotid arteries of rabbits fed with 2% cholesterol-enriched diet for 4 weeks.

In the present study, cholesterol feeding did not show any fatty streak lesion in carotid arteries as demonstrated by Red-Oil staining. It is well known that in rabbits, the aorta, the pulmonary, and carotid arteries are susceptible to atherosclerosis [21, 22]. However, in the experimental models of atherosclerosis, the influence of varying periods of cholesterol feeding on plaque formation has been demonstrated [21]. Thus, in our study, short-term (4 weeks) cholesterol feeding resulted in the functional alteration of endothelial responses (c.f. infra) without inducing plaque formation, suggesting the time dependency of the formation of lesions to cholesterol feeding. On the other hand, it has been reported that endothelial dysfunction precedes vascular wall lesions in the early phase of hypercholesterolemia [23].

The term endothelial dysfunction commonly refers to a reduced endothelium-dependent vasodilator capacity of the arteries to acetylcholine [24]. Since it is known that this type of vasorelaxation is related to the production of nitric oxide from endothelial cells, endothelial dysfunction is typically attributed to a decreased production, and/or increased catabolism of the endothelium-derived NO [24]. During the past 20 years, several studies have shown that hypercholesterolemia induces macrovascular endothelial dysfunction [22, 25]. In accordance with these findings, the results of our study showed that ACh-induced receptor-mediated, endothelium-dependent responses were impaired in the carotid artery of cholesterol-fed rabbit. In parallel, nitrite/nitrate levels of carotid artery tissues, the metabolites of nitric oxide, were decreased at the end of 4 weeks of cholesterol feeding period which suggests that hypercholesterolemia attenuates both the production and bioavailability of nitric oxide [26].

Recent studies have shown the restoration of endothelial function by statins before significant reduction of serum cholesterol levels, indicating the possibility of another/other mechanism(s), independent of cholesterol reduction [10]. These direct effects of statins on the endothelium were first defined by their ability to enhance endothelial NO production, upregulating endothelial NO synthase (eNOS) [27, 28]. In our study, consistent with previous reports, fluvastatin treatment restored the impaired relaxations to ACh in cholesterol-fed rabbits [29, 30]. Moreover, fluvastatin normalized the nitrite/nitrate levels of carotid artery tissues. These findings possibly suggest a protective role of the drug on the NO bioavailability in vascular wall [31].

As discussed above, along with the alterations of ACh-induced relaxations, the present study demonstrated that contractile responses to 5-HT were significantly enhanced in carotid arteries from high cholesterol fed rabbits without any change in pD_2_ values, pointing to the fact that high cholesterol diet did not affect the sensitivity of serotonergic receptors. Increased 5-HT-induced contractions have been reported in high-fat-diet-fed rat thoracic aorta [32] and rabbit aorta [33] and in diabetic rabbit carotid artery [17]. These enhanced contractile responses to 5-HT have been attributed to receptor-mediated or nonreceptor-mediated pathways. As seen in Figure 2, the role of non-receptor mediated contraction can be ruled out since there was no change in contractile response to KCl in carotid arteries from all groups. It has been speculated that the increased contractions to 5-HT can be related to 5-HT_2A_ upregulation in spontaneously hypertensive rats [34]. Miranda et al. reported the modulatory role of the endothelium in the constrictor response of rabbit carotid artery to 5-HT by releasing NO [17]. Thus, in our study, the increased 5-HT responses may be due to diminished NO response because of the high cholesterol diet. Indeed, as seen, Figures 4(a), 4(b), and 4(c), inhibition of NO synthesis by LNA in rings from all groups revealed that endothelial NO plays an important role in cholesterol-induced alterations in vascular serotonergic responses. Similarly, as seen in Figures 4(d), 4(e), and 4(f), inhibition of cyclooxygenase products by indomethacin did not affect 5-HT receptor-mediated responses in carotid arteries. On the other hand, fluvastatin restored the augmented contractile responses to 5-HT in carotid artery rings, as seen in Figures 3 and 4. As discussed above, the results of these findings from *in vitro* functional studies have suggested that fluvastatin may enhance endothelial NO production. However, in the present study, we did not determine the levels of nitrated proteins, that is, nitrotyrosine and eNOS expression by using any other method such as immunoblotting and western blotting. In this regard, this effect of fluvastatin should be further investigated.

In this study, while the SOD activity was increased by cholesterol feeding, the catalase activity was found to be decreased. Various studies reported different results in SOD activity in hypercholesterolemic conditions [35–39]. Thus, the activity of this enzyme may be related to the extent and duration of hypercholesterolemic diet. In our study, since fluvastatin significantly lowered the increased SOD activity by cholesterol diet, we proposed that the enhanced activity of SOD in carotid artery tissues may be related to the change in NO levels.

In conclusion, this study suggests that fluvastatin, independent of its lipid lowering properties, has a protective effect on the endothelial responses, which are compromised after cholesterol feeding.

## Figures and Tables

**Table 1:** Body weights and plasma total cholesterol levels from all groups of rabbits.

		Control group	Cholesterol group	Cholesterol + fluvastatin group
Body weight (kg)	Start	2.64 ± 0.08	2.69 ± 0.13	2.67 ± 0.07
	End	2.77 ± 0.09	2.56 ± 0.09	2.50 ± 0.07
Plasma total cholesterol (mmol/L)	Start	0.94 ± 0.15	1.30 ± 0.18	1.02 ± 0.18
	End	1.20 ± 0.13	13.82 ± 1.88^+^	9.40 ± 1.07*

^+^ *P* < 0.001, control versus cholesterol and *P < 0.01 control versus cholesterol + fluvastatin, ANOVA followed by Bonferroni *post hoc *test. Data are expressed as means of five independent measurements using five animals in each group. Data are given ± S.E.M.

**Table 2:** Antioxidant enzyme activities and total nitrite/nitrate levels in carotid artery tissues from all groups.

	Control group	Cholesterol group	Cholesterol + fluvastatin group
SOD (U/g protein)	1.56 ± 0.25	4.60 ± 0.41 ^+^	3.36 ± 0.12 *
Catalase (U/g protein)	2.13 ± 0.34	0.74 ± 0.08 ^+^	1.24 ± 0.23
Total nitrite/nitrate (nmol/mg protein)	723.90 ± 18.98	308.80 ± 1.80 ^+^	705.20 ± 7.19 *

^+^ *P* < 0.001, control versus cholesterol and *P < 0.05, cholesterol versus cholesterol + fluvastatin in SOD, and ^+^ *P* < 0.01, control versus cholesterol in catalase antioxidant enzyme activities. ^+^ *P* < 0.001, control versus cholesterol and *P < 0.001 cholesterol versus cholesterol + fluvastatin in total nitrite/nitrate (NO_2_^−^/NO_3_^−^) levels of carotid artery tissues. Data are given ± S.E.M. ANOVA followed by Bonferroni post hoc test. Data are expressed as means of five independent measurements using five animals in each group.
